# Satralizumab after inebilizumab treatment in a patient with recurrent neuromyelitis optica spectrum disorder: A case report

**DOI:** 10.1097/MD.0000000000042067

**Published:** 2025-04-04

**Authors:** Duanyang Li, Jinhao Ye, Zhaoxiang Hong, Haibing Xiao

**Affiliations:** a Department of Neurology, Neuromedicine Center, The University of Hong Kong – Shenzhen Hospital, Shenzhen, Guangdong Province, China; b Shenzhen Clinical Research Center for Rare Diseases, Shenzhen, China.

**Keywords:** immunotherapy, interleukin-6 receptor inhibitor, neuromyelitis optica spectrum disorders, satralizumab

## Abstract

**Rationale:**

Neuromyelitis optica spectrum disorder (NMOSD) comprises a group of rare and severe autoimmune inflammatory diseases affecting the central nervous system, mainly the optic nerves and spinal cord. Phase III studies have shown that the incidence of relapse is significantly reduced in aquaporin (AQP) 4 antibody-positive patients after treatment with satralizumab, a humanized monoclonal recycling antibody that blocks interleukin (IL)-6 signaling pathways, in conjunction with inebilizumab, a B-cell-depleting agent. Here, we report our experience with a patient who presented with pain associated with NMOSD.

**Patient concerns:**

A 40-year-old woman initially presented with acute thoracic myelitis. Magnetic resonance imaging revealed a demyelinating lesion in the spinal cord spanning from T2 to T10, along with enhancement and a positive serum AQP4-immunoglobulin G (IgG) titer.

**Diagnosis:**

NMOSD.

**Interventions:**

The patient initially received adequate long-term immunotherapy with inebilizumab and corticosteroids. Satralizumab was administered after treatment failure.

**Outcomes:**

The patient experienced recurrences of the disorder despite the initial immunotherapy, including pain and immobility from neurological dysfunction. Furthermore, her serum AQP4-IgG titer remained elevated (1:320), her B-cell proportion remained at 0, and her symptoms were not adequately relieved. She was then administered satralizumab, after which her serum AQP4-IgG and IL-6 levels decreased, the radiological appearance of spinal cord demyelination improved, her pain and other symptoms were alleviated, and her neurological function gradually recovered.

**Lessons:**

In patients with clinical episodes of NMOSD that recur despite treatment with a B-cell-depleting agent, satralizumab may help alleviate myelitis-associated pain. Further investigations are warranted to establish IL-6 as a therapeutic target for the treatment of neuropathic pain, and may help address the unmet medical need in the management of NMOSD-associated neuropathic pain. As exemplified by the present case, individualized management, and therapy for patients with NMOSD are essential. Our case report provides new ideas for the management of patients with refractory NMOSD and patients with subsequent severe neuropathic pain.

## 1. Introduction

Neuromyelitis optica spectrum disorder (NMOSD) comprises a group of rare and severe autoimmune inflammatory diseases that target the central nervous system and affect mainly the optic nerves and spinal cord.^[1]^ Rituximab, a monoclonal antibody against CD20, is commonly used to treat this disorder.^[2,3]^ In addition, recent phase III studies have shown that, compared with that for those given placebo, the incidence of relapse is significantly lower for aquaporin (AQP) 4 antibody-positive patients treated with satralizumab,^[4]^ an interleukin (IL)-6 receptor inhibitor, in conjunction with inebilizumab, an antibody against CD19 B cells,^[5]^ and eculizumab, an antibody blocking the activation of complement protein 5.^[3]^ Here, we describe our experience with an NMOSD patient who continued to experience disease recurrences despite treatment with inebilizumab. Once satralizumab was initiated, the patient gradually improved over several months.

Satralizumab is a type of humanized anti-IL-6 receptor monoclonal recycling antibody. Some clinical trial results^[3]^ have suggested that satralizumab results in a lower risk of relapse than placebo among patients who are AQP4-immunoglobulin G (IgG)-seropositive. Further clinical and nonclinical investigations are needed to establish IL-6 as a therapeutic target for the treatment of neuropathic pain associated with neuroimmunological disorders such as NMOSD.

## 2. Case presentation

A 40-year-old Chinese woman presented to a local hospital (January 2023) because of numbness and pain in her waist and back. She rated her back pain as 7 on a 10-point numeric rating scale (NRS). Magnetic resonance imaging (MRI) revealed a demyelinating lesion in the spinal cord spanning from T2 to T10 along with enhancement, and the patient was positive for serum AQP4-IgG (1:100; reference negative). NMOSD was diagnosed, and intravenous immunoglobulin (IVIG; 0.4 g/kg/day) was administered for 5 days, which relieved her symptoms to some degree. However, the numbness persisted, and her back pain was rated 6 on the NRS at discharge. She then began to receive regular infusions of inebilizumab as long-term immunotherapy.

**Figure 1.**
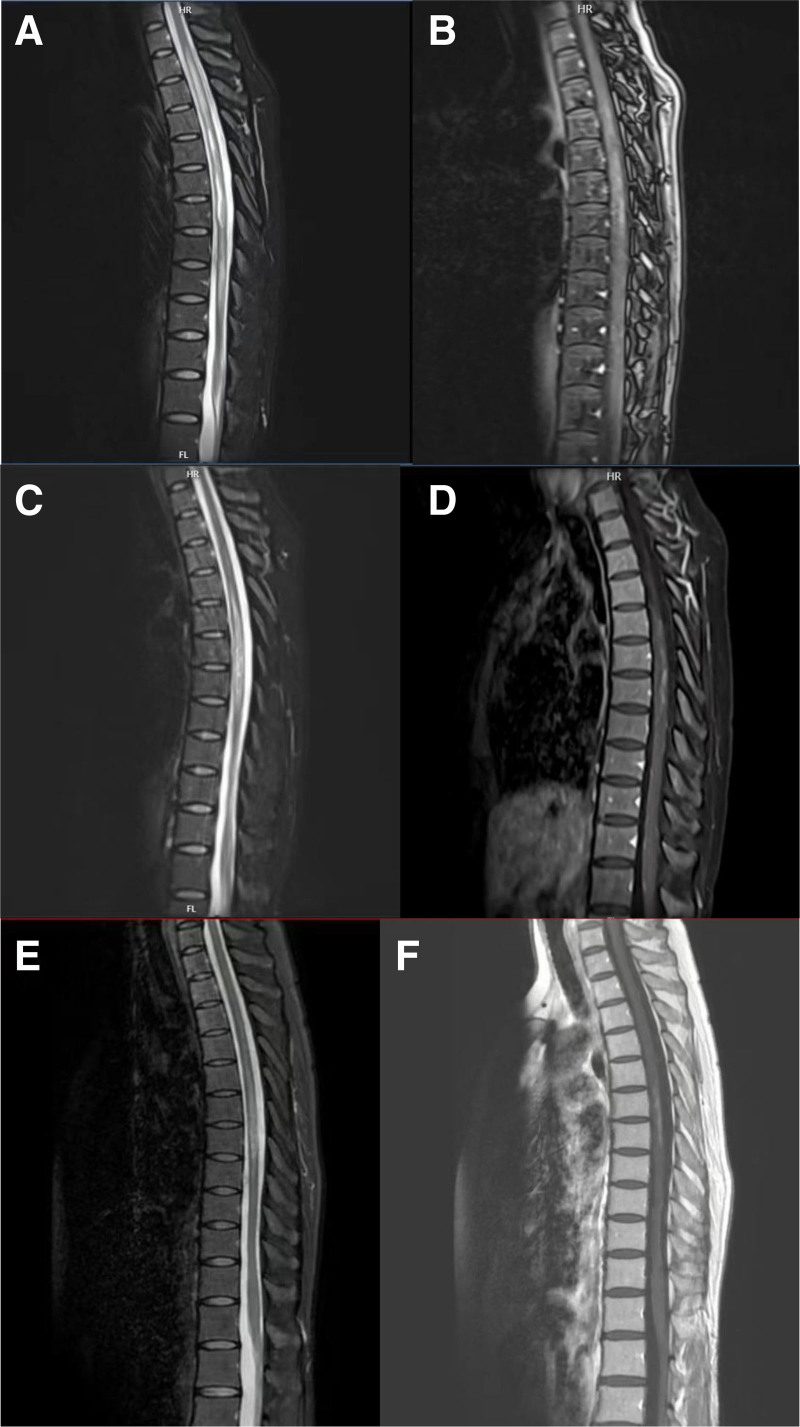
Sagittal magnetic resonance images of the thoracic spine: T2-weighted and T1-weighted imaging with contrast. (A and B) A long demyelinating lesion exhibiting contrast enhancement is seen from T2 to T10, 2 months after her initial presentation. (C and D) Images 4 months later revealing demyelination from T4 to T8. (E and F) Images acquired almost 1 year after diagnosis revealing demyelination from T4 to T7.

Two months later, she again developed numbness, weakness, and stiffness in her left lower limb and could not walk without assistance. Her back pain increased to 7 on the NRS. Repeat MRI revealed enhancement of the demyelinating lesion (Fig. 1A, B), and her serum AQP4-IgG titer significantly increased (1:320). She was admitted to our hospital, and high-dose methylprednisolone pulse therapy (500 mg/day for 3 days, then 250 mg/day for 3 days) was initiated. Although her left lower limb strength recovered somewhat, she complained of residual paroxysmal spasms and pain in that limb.

Four months later (July 2023), she was admitted to another hospital because of back pain (grade 7 on the NRS), bilateral calf numbness, and urinary retention. Another MRI revealed an active demyelinating lesion extending from T4 to T8 (Fig. 1C, D). Methylprednisolone pulse therapy was administered again along with IVIG (0.4 g/kg/day) for 3 days, which did not improve her symptoms. Plasma was exchanged 7 times subsequently. Despite this treatment, her serum AQP4-IgG titer remained elevated (1:320), her B-cell proportion remained zero, and her symptoms were not adequately relieved.

For further treatment, the patient was hospitalized again 1 month later (August 2023) at the University of Hong Kong – Shenzhen Hospital because of her inability to walk independently and back pain (grade 7 on the NRS). On a physical neurological examination, she had grade 4 strength in both lower limbs and decreased vibratory sensation below the knees. Babinski testing was positive bilaterally, and her score on the Expanded Disability Status Scale (EDSS; range, 0–10) was 6.5 (she required 2 walking aids [1 for each side] to walk 20 m without rest). Her serum AQP4-IgG titer was 1:100, and her serum IL-6 level was 32.8 pg/mL (reference range, 0.5–8.0 pg/mL). Satralizumab was administered 3 times between August 2023 and September 2023, and no allergic reactions or chronic infections occurred. Her back pain had improved notably by discharge in August 2023 (grade 5 on the NRS).

A month later, she was able to walk 20 m without assistance but had a wide-based gait and complained of back pain (graded 4 on the NRS). Muscle strength in both lower limbs was grade 4+, and her EDSS score was 5.0 (she could walk 200 m independently without rest). The serum IL-6 level was 20.3 pg/mL. She was admitted again, and a fourth injection of satralizumab was administered. No allergic reaction or chronic infection occurred.

The patient was hospitalized again 6 weeks later (December 2023) with chest and back numbness and an unstable gait; however, she could walk more than 500 m independently. Her back pain had decreased to 2 on the NRS. On physical examination, her muscle strength was grade 5 throughout, but she walked with a slightly wide-based gait. Her EDSS score was 4.0 (she could walk 500 m independently without rest). During hospitalization, repeated MRI revealed a decrease in the area of demyelination and decreased enhancement (Fig. 1E, F). Her serum IL-6 level was 16.2 pg/mL. She received a 5th injection of satralizumab with immunoglobulin (0.4 g/kg/day) for 5 days. At discharge, her back pain was relieved. The following month, her serum AQP4-IgG titer decreased to 1:32 (reference value was negative), and her serum IL-6 level had decreased to 6.7 pg/mL. She received a 6th injection of satralizumab, and her EDSS score decreased to 2.0 (minimal disability).

We have continued to revisit this patient to pay close attention to the development of and changes in the disease. The patient’s condition has been stable (EDSS score 2), the pathological neuropathic pain has resolved completely, and there has been no recurrence of NMOSD since the regular injection of satralizumab. The successful outcome of this treatment indicates that anti-IL-6 therapies for ameliorating neuropathic pain as a primary endpoint in clinical trials are warranted and may help address the unmet medical need in the management of neuropathic pain associated with NMOSD.

In the present case, we found that the patient’s symptoms were recurrent despite B cells being present at a low level. This suggested the need to switch our treatment plan to an agent targeting a different pathway. Notably, the patient’s neuropathic pain was significantly reduced after the regular use of satralizumab, a humanized monoclonal recycling antibody that can bind to membrane-bound and soluble IL-6 receptors, thereby blocking IL-6 signaling pathways. Both clinical and nonclinical trials^[10]^ have shown that anti-IL-6 therapies can be used for the treatment of neuropathic pain.

**Figure 2.**
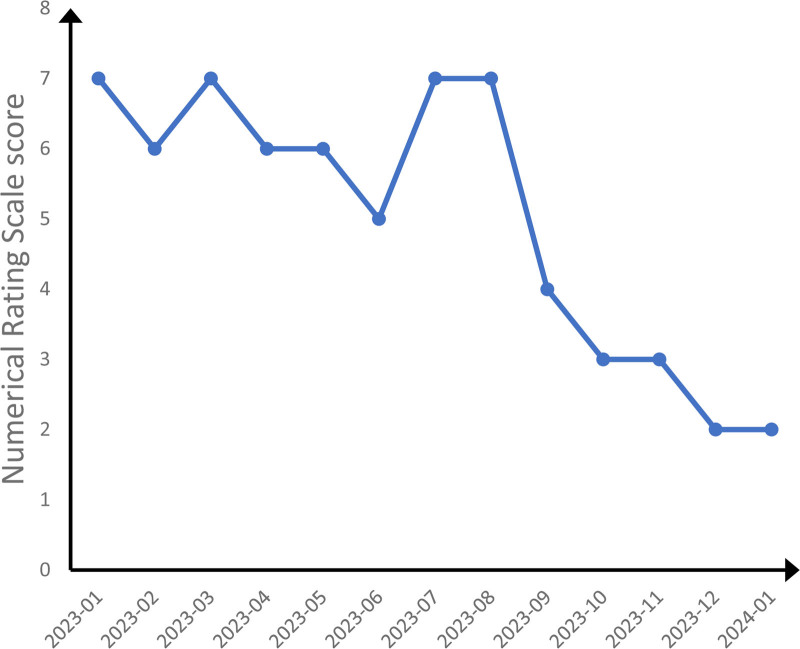
Back pain, as rated on the numerical rating scale from the time of diagnosis (January 2023) to the most recent follow-up (January 2024).

**Figure 3.**
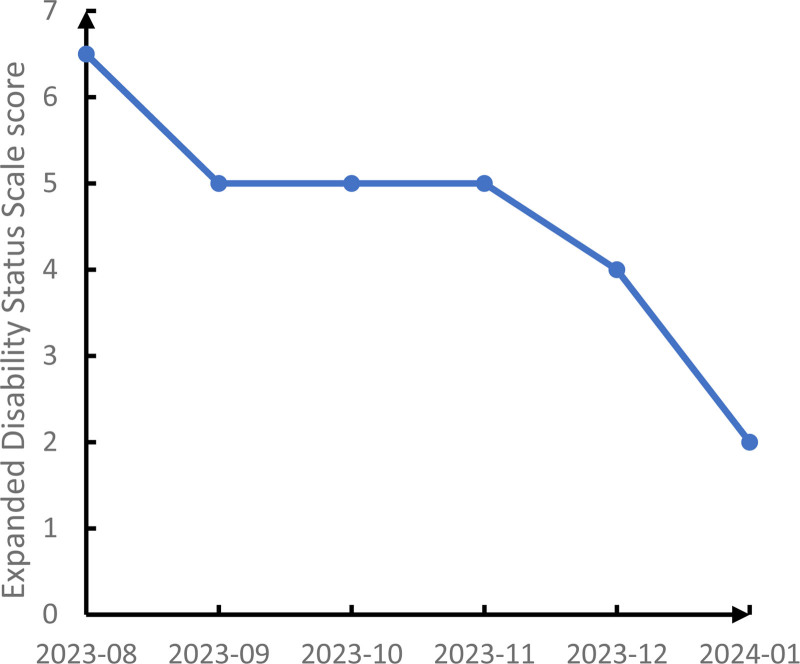
Expanded Disability Status Scale scores over time after satralizumab administration was initiated.

**Figure 4.**
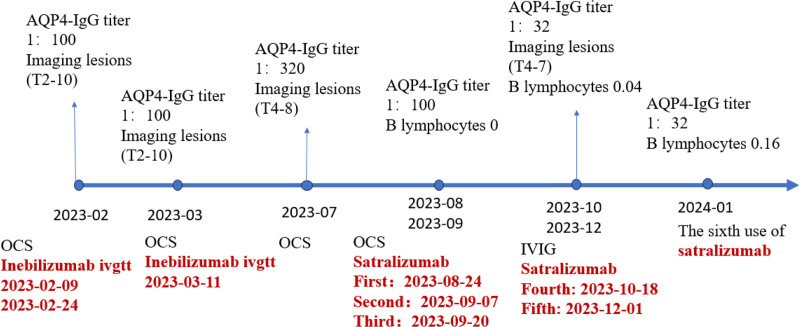
Timeline of laboratory and imaging findings and treatment. AQP4-IgG = aquaporin 4-immunoglobulin G; IVIG = intravenous immunoglobulin; OCS = oral corticosteroid.

Once satralizumab treatment was initiated and readministered at the time of symptom exacerbation, the patient’s clinical symptoms, and the radiological appearance of spinal cord demyelination improved. No allergic reactions or chronic infections occurred. Her B lymphocytes were maintained at a low level throughout the course of treatment, because the goal for NMOSD patients is 0. Her NRS scores for back pain and EDSS scores over time are shown in Figures 2 and 3. Figure 4 shows a timeline of the laboratory and imaging findings and treatments.

## 3. Discussion

NMOSD is an inflammatory demyelinating autoimmune disease of the central nervous system that involves mainly the optic nerve and spinal cord^[1]^; brain and brainstem involvement have also been reported.^[6]^ The main clinical symptoms are acute transverse myelitis and optic neuritis. Repeated acute attacks and the presence of AQP4-IgG antibodies are the typical characteristics of NMOSD and are associated with high rates of recurrence rate and disability.^[7]^ Autoantibodies against AQP4 are present in more than 80% of NMOSD patients and play a central role in disease pathogenesis and an increased understanding of the pathophysiology of AQP4-IgG-positive NMOSD has led to the development of targeted therapeutic approaches. Long-term immunosuppression may be used in NMOSD patients to reduce the incidence of recurrence, decrease disability, and improve outcomes and quality of life.^[3]^ In addition to nonspecific immunosuppressants, such as oral glucocorticoids, azathioprine, or mycophenolate mofetil, more tailored therapeutic approaches have been developed that specifically target the pathophysiology of the disease. A variety of monoclonal antibodies have been developed to prevent recurrence, including those that block the activation of complement protein 5 (eculizumab and ravulizumab),^[8]^ clear B cells (inebilizumab and rituximab),^[9]^ and block the IL-6 receptor (tocilizumab and satralizumab).^[10]^ These antibodies have a favorable side effect profile and significantly lower the recurrence rate.^[11]^ In the near future, immunotherapies may also include antibodies against the neonatal Fc receptor.

Our patient in this case underwent treatment with satralizumab after responding poorly to other long-term immunotherapies, such as oral methylprednisolone and inebilizumab. Inebilizumab, like rituximab, is a monoclonal antibody that depletes B cells^[5]^; however, inebilizumab targets CD19 rather than CD20 and is effective only in patients positive for AQP4-IgG.^[12]^ In previous reports, traditional B-cell-depleting agents have shown to be effective in preventing NMOSD recurrence. B cells play an important role in the pathophysiology of NMOSD. ^[13]^ Memory B cells self-react with AQP4 and thus differentiate into plasma blasts and mature plasma cells that produce AQP4-IgG.^[13]^ However, serum AQP4-IgG titers do not significantly decrease in all NMOSD patients treated with a B-cell-depleting agent; therefore, some long-lived plasma cells that cannot be eliminated are likely involved via other mechanisms, such as antigen presentation to activated T cells and the secretion of proinflammatory cytokines.^[14]^ In our patient, after inebilizumab treatment, B lymphocytes were not detected in the blood; however, her AQP4-IgG titer remained elevated and MRI revealed active demyelination in the spinal cord. It is possible that long-lived plasma cells^[15]^ are present in the bone marrow and cannot be eliminated by inebilizumab or detected by conventional methods. These long-lived plasma cells continuously secrete antibodies, which can result in recurrent disease episodes. The disease was subsequently controlled gradually after satralizumab was introduced, suggesting that satralizumab may inhibit long-term plasma cell activity.

Satralizumab is a humanized monoclonal antibody that targets the IL-6 receptor^[4]^ and is effective in AQP4-IgG-seropositive NMOSD patients.^[10]^ IL-6 not only plays an essential role in the production of AQP4-IgG,^[16]^ but also increases the permeability of the blood–brain barrier to help it enter the brain.^[17]^ Thereby, satralizumab can reduce inflammation-related injury in the central nervous system by blocking the IL-6 pathway.^[18]^ Although our patient still experienced several episodes of symptoms while receiving inebilizumab, her symptoms gradually improved within several months of initiating satralizumab. In this case, the clinical diagnosis and treatment of the patient are important for guiding the formulation of our long-term treatment plans for patients with recurrent NMOSD.

IL-6 is an inflammatory cytokine that plays an essential role in neuropathic and inflammatory pain.^[19]^ In NMOSD patients with spinal cord lesions, pain is a common complaint.^[20]^ As an IL-6 receptor blocker, satralizumab may help relieve this pain in NMOSD patients.^[10]^ Neuropathic pain is common in such patients, and 50% or more patients with NMOSD experience chronic neuropathic pain. However, the currently available medications for the management of neuropathic pain have limited effectiveness in patients with NMOSD, and there is an unmet medical need to identify new therapies for the management of chronic neuropathic pain in these patients. Thoracic myelitis was the primary clinical manifestation in our patient, who presented with recurrent back and left lower limb pain. Satralizumab demonstrated a clear beneficial effect on reducing her reported level of pathological pain. This effect may provide us with new ideas for treating residual neuropathic pain in NMOSD patients.

Individualized management and therapy in NMOSD patients are essential. They should be considered based on this case, previous broad immunosuppression in NMOSD and now shifting to tailored treatments that promise to be more efficient. Individualized therapy must be expanded to choose more treatments, including newly-developed biologics, because current effective treatments for acute episodes of NMOSD result in complete recovery in approximately 30% of episodes only. The disease recurred many times in the present patient, and these dangerous recurrences can lead to patient disability or even death. Therefore, we suggest that physicians should not necessarily be restricted to the use of only first- or second-line traditional drugs for NMOSD. Future treatment should be based on patient responses, including the efficacy, safety, and tolerability of these initial treatments, to inform the use of drugs with different curative mechanisms. Taking our patient in this case as an example, in the future clinical diagnosis and treatment of NMOSD, it is necessary to make an individualized treatment plan as soon as possible to control the recurrence of the disease in a timely and effective manner.

## 4. Conclusion

Understanding of NMOSD continues to improve, and various new kinds of treatments for NMOSD have been developed. However, the efficacy of the newly-developed therapeutics remains to be fully confirmed because of a lack of extensive clinical data. Satralizumab may help alleviate myelitis-associated pain, and thus have a role in treating cases of NMOSD where symptoms recur despite treatment with a B-cell-depleting agent. Further investigations to establish IL-6 as a therapeutic target for the treatment of neuropathic pain are warranted, and may help address the unmet medical need for management of NMOSD-associated neuropathic pain. The present case exemplifies the need for individualized management and therapy for patients with NMOSD.

## 5. Limitations

This case report has several limitations that warrant consideration. First, as a single-case study, the findings may lack generalizability to broader populations because of the unique clinical characteristics and individual variability inherent to the presented case. Second, the retrospective nature of the data collection introduces a potential recall bias and incomplete documentation of certain clinical parameters. Finally, while this report highlights a new therapeutic approach to patients with NMOSD-associated neuropathic pain, further multicenter studies with larger sample sizes are needed to validate these observations and explore the underlying mechanisms.

## Acknowledgments

We thank Robin James Storer, PhD, from Liwen Bianji (Edanz) (https://www.liwenbianji.cn) for editing the English text of drafts of this manuscript and helping to draft the abstract.

## Author contributions

**Conceptualization:** Zhaoxiang Hong, Haibing Xiao.

**Investigation:** Zhaoxiang Hong.

**Resources:** Duanyang Li.

**Supervision:** Haibing Xiao.

**Writing – original draft:** Duanyang Li, Zhaoxiang Hong.

**Writing – review & editing:** Jinhao Ye, Zhaoxiang Hong, Haibing Xiao.
